# A single-bridge interleaved three-level LLC resonant converter with current sharing capability for fuel cell system

**DOI:** 10.1038/s41598-024-67456-1

**Published:** 2024-07-17

**Authors:** Qianqi Zhao, Xu-Feng Cheng, Yazhuo Li, He Li, Dianlong Wang, Chenyang Liu, Yong Zhang

**Affiliations:** 1 School of Mechanical Engineering, Hebei University of Science and Technology, Shijiazhuang, 050018 People’s Republic of China; 2 School of Mechanical Engineering, Yanshan University, Qinhuangdao, 066004 People’s Republic of China; 3 College of Marine Electrical Engineering, Dalian Maritime University, Dalian, 116026 People’s Republic of China; 4 School of Mechanical Engineering, Shanghai Jiao Tong University, Shanghai, 200240 People’s Republic of China

**Keywords:** Energy science and technology, Engineering, Electrical and electronic engineering

## Abstract

This paper propose a wide gain single-bridge interleaved three-level LLC resonant converter with current sharing capability. This novel converter offers numerous advantages, including low cost, low current ripple, reduced voltage and current stress, widely gain range, high efficiency, good current sharing capability and versatile application scalability. Utilizing the three-level inverter + full-wave rectifier LLC converter as a representative case, the paper conducts an in-depth analysis and research on the proposed method. Finality, a 600 W experimental prototype was constructed and tested. Experimental results reveal that the proposed converter exhibits lower current ripple and a broader gain range. Moreover, the converter shows good current sharing capability (with a resonant element tolerance of 10%, the current error between the two phases does not exceed 12%) and high efficiency (peaking at 95.8%).

## Introduction

**Figure 1:**
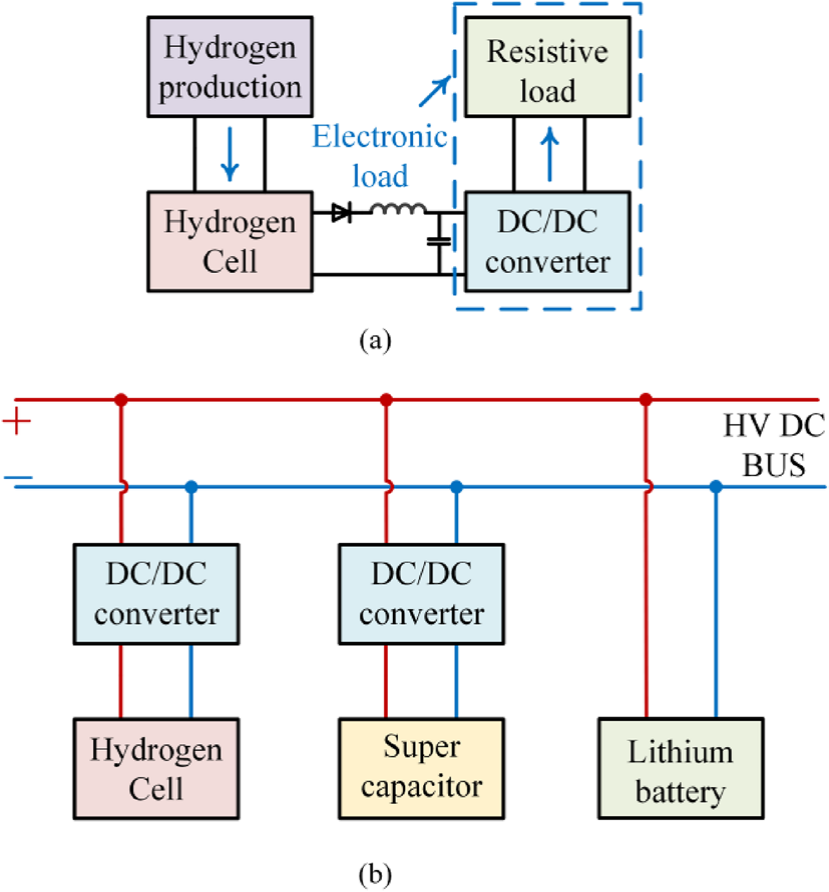
The application of DC/DC converter in fuel cell system. (**a**) Fuel cell activation device. (**b**) Fuel cell energy storage system. (The activation device of the fuel cell consists of a DC / DC converter and a resistive load. The DC / DC converter can discharge the fuel cell for the first time according to the specified current trajectory, power trajectory or load trajectory. And the fuel cell energy storage system includes a fuel cell, a lithium battery and a supercapacitor. The fuel cell and the supercapacitor are connected to the DC bus through a DC / DC converter, and the lithium ion battery is directly connected to the DC bus).

With the global increasing demand for clean energy and sustainable development, fuel cells have shown great potential in the field of clean energy due to their advantages of zero pollution, low noise, high efficiency and high reliability^1–3^. This has garnered attention worldwide. At present, fuel cells are widely used in various fields, including electric vehicles, fuel cell grid-connected power generation systems, aerospace, etc.^4–6^. However, two problems need to be solved in the manufacture and use of fuel cells. First, the discharge and energy storage characteristics of fuel cells are not optimal when they are manufactured, so they need to be activated. The so-called activation refers to the initial high-current discharge of fuel cells according to certain rules. As shown in Fig. 1a, the fuel cell activation system is described. The activation device in the figure comprises a DC/DC converter and a resistive load. The DC/DC converter facilitates the initial discharge of the fuel cell along a specified current, power, or load trajectory. According to literatures^7,8^, the discharge current trajectory is set to be stepped current profile to activate the fuel cell, the maximum current density is increased 14.5%. In^9^, a fast activation method using rectangular pulse current is proposed. This method greatly shortens the activation time of fuel cell. Among these activated waveforms, stepped current profile can obtain the maximum energy storage performance and the rectangular pulse current can obtain the maximum power performance. This shows the important role of DC/DC converters in fuel cell activation.

Another problem that needs to be solved in the use of fuel cells is its output soft characteristics, that is, its output voltage is not constant. Therefore, fuel cells require a DC/DC converter as a power regulator when in use. Figure 1b shows the circuit structure of fuel cell applied to fuel cell vehicles or fuel cell power generation systems. The figure contains fuel cells, supercapacitors and lithium batteries. Electricity generated by the fuel cell is first stored in the supercapacitor, then transferred to the lithium battery, thus forming a composite energy storage system for automotive and grid power applications. In summary, the DC/DC converter is the most critical component in the manufacture and use of hydrogen fuel cells. The DC/DC converter with excellent performance can promote the progress of the fuel cell industry.

The DC/DC converters used in fuel cells are divided into isolated and non-isolated. Among them, the main characteristics of the non-isolated DC/DC converter are simple structure, easy control, and no electrical isolation. At present, In^10^, a non-isolated DC/DC converter for hydrogen fuel cell electronic load is proposed. While in^4^, a DC/DC converter as a power regulator of fuel cell is proposed, however, the converter has no electrical isolation, the step-up curve is steep and difficult to control, and the power that a single converter can achieve is low. Compared with the non-isolated DC/DC converter, the most important feature of the isolated DC/DC converter is its electrical isolation, so it is more suitable for high-power applications.

The LLC resonant isolated DC/DC converter is widely adopted in fuel cell energy storage systems and activation^11–15^, it stands out for its easy of control, simple structure, low electromagnetic interference, fast response, high reliability and high efficiency. However, the conventional single-phase LLC resonant converter exhibits limitations with its narrow output current range and high ripple, falling short of meeting the specific demands for DC/DC converters in the activation and use of fuel cells.

The application of interleaved parallel technology can significantly solve the problem of high output current ripple of LLC resonant converter^16,17^. However, in the context of the LLC resonant converter, the parameters of its resonant elements wield considerable influence over its gain. The multi-phase interleaved parallel technology will introduce multiple sets of resonant tanks. To uphold reliability, it becomes essential to ensure uniformity in the parameters of the resonant elements among the resonant tanks in each phase. However, in actual manufacturing, due to the errors in production process, it is inevitable that the resonant elements between different phases have tolerances, which causes an imbalance of current between the phases, which will adversely affect the life and heat dissipation of the converter. Consequently, the adoption of a current sharing method becomes essential in a multi-phase parallel LLC resonant converter.

Current mainstream current sharing methods are mainly divided into active and passive two type. Among them, the active current sharing method refers to the use of active devices, combined with control strategies to achieve uniform distribution of load current between phases^18^. proposed a method of compensating the impedance mismatch between resonant networks with variable resonant inductors with output current as the control variable. Similarly^19,20^, used variable capacitors. However, these methods essentially monitor the current in the resonant tank in real time through the sensor, which not only requires high sampling accuracy of the sensor, but also adversely affects the cost and reliability of the converter. In^21^, a controllable auxiliary voltage source is formed, by integrating a transformer and a DC/DC converter into each phase, which is utilized to adjust the equivalent input voltage of the resonant network in each phase, facilitating the achievement of current sharing between phases. However, this method introduces an auxiliary converter, which is not favorable for reducing the cost and size of the overall converter. In^16^, a method of current sharing using virtual controllable voltage sources (VCVSs) is proposed. The specific principle is to use the auxiliary winding of the transformer to couple to the resonant slot of the other phase, and then adjust the phase between the two phases through the control strategy to achieve current sharing. However, this method poses challenges in terms of control, which hampers efforts to enhance the reliability of the converter. In general, the active current sharing method is both costly and difficult to control, posing obstacles to enhancing the overall reliability of the system.The passive current sharing method typically employs passive devices like capacitors, inductors, and transformers to achieve current sharing. This method offers advantages such as ease of implementation, low loss, and cost-effectiveness^22^. Proposes a current sharing method for co-resonant inductors. Similarly^23,24^, Uses a co-resonant capacitor^25^. Proposed a magnetic coupling-current balancing (MC-CB) cell, which is connected between the two-phase LLC resonant tanks to achieve current sharing^26^. Proposed a current sharing method based on the grouping of auxiliary windings on the secondary side of the transformer. However, due to the introduction of auxiliary windings on the secondary side of the transformer, it is difficult to accurately control the leakage inductance of each winding, which will lead to the mismatch of the leakage inductance on the secondary side and adversely affect the current sharing effect. On this basis^27^, solved the problem of secondary side leakage inductance mismatch by changing the connection mode of the secondary side winding of the transformer. Although the passive current sharing method mentioned above^22–27^ is simple in control and easy to implement, it is not suitable for staggered parallel structures.

In order to solve the above problems and meet the requirements of hydrogen fuel cell applications for DC/DC converters, this paper proposes a single bridge interleaved three-level LLC converter with current sharing capability, combining the interleaving technology of^17^ and the current sharing technology of^26^. The current sharing method proposed in^26^ uses auxiliary windings on the secondary side of transformers to connect the secondary sides of two transformers in series to achieve current sharing, which has the advantages of simple structure and easy implementation. However, in this structure, the phase of the secondary current between the two phases of the converter is always the same, so it cannot be applied in the interleaving field. On this basis, this article symmetrically connects the secondary sides of two transformers with the auxiliary winding on the secondary side of the transformer. This structure can achieve the current sharing effect in^26^. At the same time, because the auxiliary winding adopts a symmetrical series connection method, the phase difference of the secondary side currents of the two phases of the converter is 180 degrees, thereby achieving interleaving and reducing the output current ripple. In addition, the two resonant cavities of the proposed converter share a primary inverter, thereby reducing the cost and volume of the converter. Finally, a PWM + PFM hybrid modulation strategy is adopted to achieve a wide gain range. Therefore, the proposed converter is suitable for applications that require a wide gain range, high power, and low ripple conditions. This includes applications such as fuel cell activation, electric vehicle charging stations, battery energy storage, and fuel cell systems. Overall, this article has four contributions:

- In comparison to the conventional two-phase parallel three-level LLC converter, four switches are saved and the cost of the converter is reduced.
- The amplitude of the output current ripple is halved and the frequency is doubled, thus reducing the demand for filter capacitors.
- The wide gain range of the converter is realized by using PFM + PWM hybrid modulation strategy.
- The current balance between the phase is achieved, and no auxiliary components and controls are added.

## Operation principle

**Figure 2:**
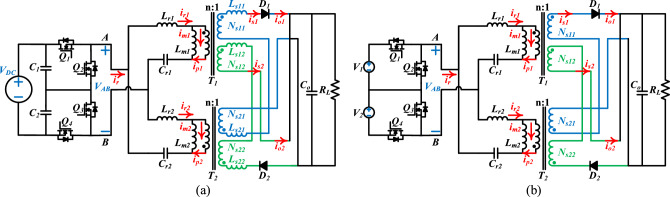
Proposed converter. (**a**) The topology structure. (**b**) The equivalent circuit. (The proposed converter consists of one voltage source, two voltage divider capacitors, four MOSFETs, two sets of resonant cavities, two transformers, two diodes and one resistive load).

Figure 2a shows the proposed converter, Figure 2b is the equivalent simplified circuit. The converter consists of two input voltage dividing capacitors (*C*_*1*_, *C*_*2*_), four switches (*Q*_1_, *Q*_*2*_, *Q*_*3*_, *Q*_*4*_), two symmetrically interleaved resonant tanks (*L*_*r1*_, *L*_*m1*_, *C*_*r1*_, *L*_*r2*_, *L*_*m2*_, *C*_*r2*_), two transformers with auxiliary windings (*T*_*1*_, *T*_*2*_), two rectifier diodes (*D*_*1*_, *D*_*2*_) and a filter capacitor (*C*_*o*_). *V*_*DC*_ is the input voltage, *R*_*L*_ is the load resistance, where *C*_*1*_ = *C*_*2*_. The two resonant tanks of the converter share a primary side. The secondary winding groups of the two transformers are composed of two identical windings *N*_*s11*_, *N*_*s12*_ and *N*_*s21*_, *N*_*s22*_ respectively. Winding *N*_*s11*_ from the first phase and winding *N*_*s21*_ from the second phase are symmetrically connected in series and connected to the rectifier circuit of phase 1, thus forming the power transmission path of phase 1. Symmetrically, the phase 2 is also the same. The two phases of the converter are combined at the output end.

### Operational modal

**Figure 3:**
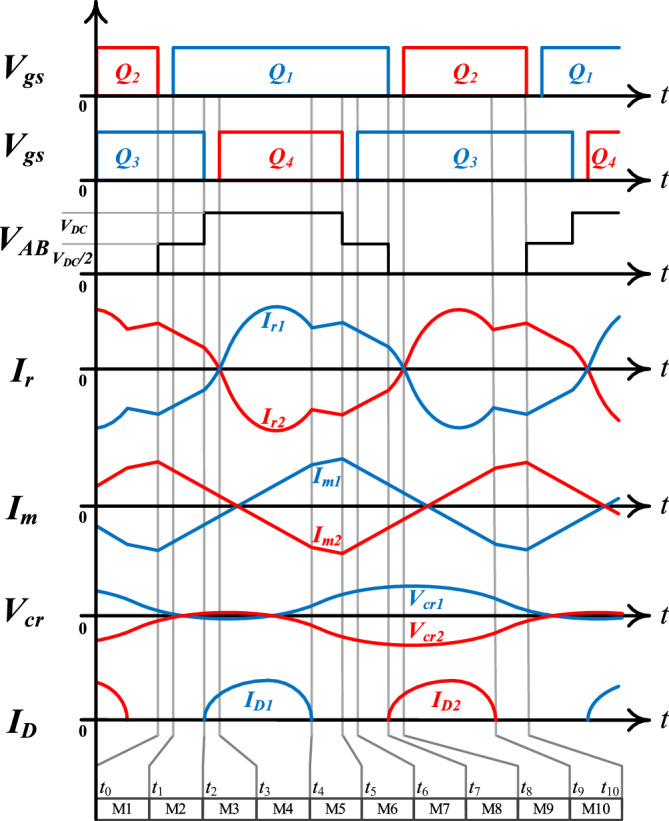
The steady-state waveform of the proposed converter (The *x*-axis is time *t*, and the *y*-axis is the key parameter of the proposed converter. At the same time, according to the working principle of the proposed converter, the diagram divides the working modes of the converter into 10 kinds in one cycle).

**Figure 4:**
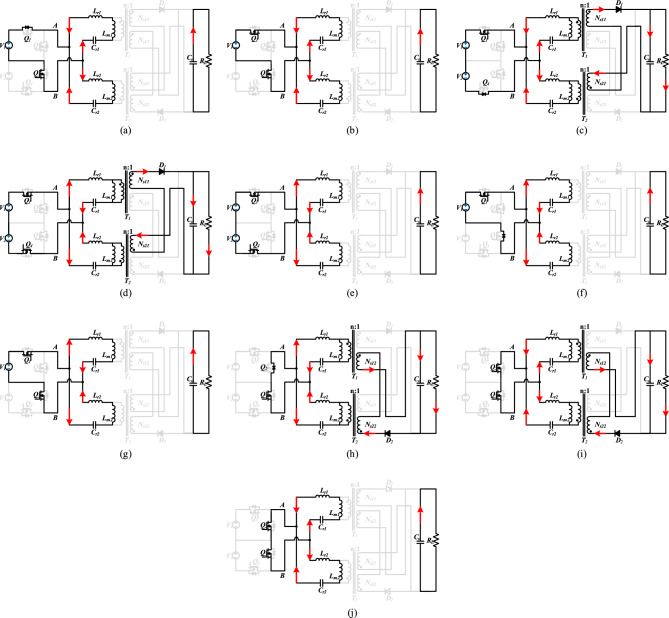
Operating intervals (According to the time sequence, the principle of the proposed converter in different working modes in one cycle is given respectively).

Figure 3 shows the driving signal of *Q*_*1-4*_ and the key steady-state waveform of the converter. It can be seen that *Q*_*1*_, *Q*_*2*_ and *Q*_*3*_, *Q*_*4*_ are complementary to each other in a certain dead time, where the duty cycle of *Q*_*1*_ and *Q*_*3*_ is *d*, the duty cycle of *Q*_*2*_ and *Q*_*4*_ is *1-d*, and the phase of *Q*_*3*_ lags behind* Q*_*1*_ by 180 degrees. When *d* > 0.5, the proposed converter operates in three-level mode. In this mode, the operating modes of the converter can be divided into 10 types, and Fig. 4 shows the equivalent circuits of these 10 operating modes. In addition, when *d* = 0.5, the proposed converter operates in a two-level mode. Compared with the three-level mode, the two-level mode does not have *M1*, *M2*, *M6* and *M7* in one cycle, and the remaining operating modes are the same as the three-level mode. Since the two phases of the converter are symmetrically interleaved, the following analysis process is given with phase 1 as an example.

*Interval M1 (t*_*0*_*-t*_*1*_, Figure 4a): In this mode, *Q*_*3*_ is in the conduction state, and *Q*_*2*_ turns off at time *t*_*0*_. The primary current is freewheeling through the parasitic diode of *Q*_*1*_, *V*_*AB*_ increases from 0 to *V*_*DC*_ / 2, and *i*_*r1*_ is negative. Since *L*_*m1*_ participates in the resonance, *i*_*m1*_ is equal to *i*_*r1*_ and rises linearly. The differential equations of *i*_*r*1_ and *i*_*m1*_ are :

1 $$\frac{{di_{r1} }}{dt} = \frac{{di_{m1} }}{dt} = \frac{{V_{DC} - V_{cr1} }}{{2\left( {L_{m1} + L_{r1} } \right)}}$$

*Interval M2 (t*_*1*_*-t*_*2*_, Figure 4b): At time *t*_*1*_, *Q*_*1*_ achieves ZVS conduction. In this mode, the resonant tank is in the ternary resonant state, and *V*_*AB*_ remains *V*_*DC*_ / 2. The load is powered by *C*_*o*_. The calculation formula of the differential equations of *i*_*r1*_ and *i*_*m1*_ is the same as that of *M1*.

*Interval M3 (t*_*2*_*-t*_*3*_, Figure 4c): At time *t*_*2*_, *Q*_*3*_ is turned off. In this mode, *Q*_*1*_ continues to be on, and the primary side current flows through the parasitic diode of *Q*_*4*_. *V*_*AB*_ rises from *V*_*DC*_ / 2 to *V*_*DC*_, and *i*_*r1*_ is negative. *L*_*m1*_ is clamped at *nV*_*o*_ (*V*_*o*_ is the output voltage), and *i*_*m1*_ rises linearly. On the secondary side, *D*_*1*_ is turned on, and *I*_*D1*_ begins to rise from zero. *N*_*S11*_, *N*_*S21*_ and *D*_*1*_ constitute the power transmission path of the converter. The differential equations of *i*_*r1*_ and *i*_*m1*_ are:

2 $$\frac{{di_{r1} }}{dt} = \frac{{V_{DC} - V_{cr1} }}{{L_{r1} }} - \frac{{8n^{2} i_{R} R_{L} }}{{\pi^{2} L_{r1} }}$$

3 $$\frac{{di_{m1} }}{dt} = \frac{{8n^{2} i_{R} R_{L} }}{{\pi^{2} L_{m1} }}$$

where *n* is the ratio of the transformer, *i*_*R*_ = *i*_*r1*_-*i*_*m1.*_

*Interval M4 (t*_*3*_*-t*_*4*_, Figure 4d): In this mode, *Q*_*1*_ is in the conduction state. At time *t*_*3*,_ *Q*_*4*_ achieves ZVS conduction. *V*_*AB*_ is *V*_*DC*_, and *i*_*r1*_ is positive. *L*_*m1*_ is clamped at *nV*_*o*_, and *i*_*m1*_ linear rise. On the secondary side, *N*_*S11*_, *N*_*S21*_ and *D*_*1*_ constitute the power transmission path of the converter. The differential equations of *i*_*r1*_ and *i*_*m1*_ are the same as those of *M3*.

*Interval M5 (t*_*4*_*-t*_*5*_, Figure 4e): In this mode. *Q*_*1*_ and *Q*_*4*_ are in conduction state. *V*_*AB*_ remains *V*_*DC*_, and *i*_*r1*_ is positive. At time *t*_*4*_, *D*_*1*_ realizes ZCS shutdown, *i*_*m1*_ is equal to *i*_*r1*_, *L*_*m1*_ participates in the resonance, and the load energy is provided by the *C*_*o*_. The differential equations of *i*_*r1*_ and *i*_*m1*_ are:

4 $$\frac{{di_{r1} }}{dt} = \frac{{di_{m1} }}{dt} = \frac{{V_{DC} - V_{cr1} }}{{L_{m1} + L_{r1} }}$$

*Interval M6 (t*_*5*_*-t*_*6*_, Figure 4f): In this mode. *Q*_*1*_ is in conduction state. At time *t*_*5*_, *Q*_*4*_ is turned off. The primary side current flows through the parasitic diode of *Q*_*3*_, *V*_*AB*_ decreases from *V*_*DC*_ to *V*_*DC*_ / 2, and *i*_*r1*_ is positive. The resonant tank is in a ternary resonant state. The differential equations of *i*_*r1*_ and *i*_*m1*_ are the same as those of *M1*.

*Interval M7 (t*_*6*_*-t*_*7*_, Figure 4g): In this mode, *Q*_*1*_ remains on. At time *t*_*6*_, *Q*_*3*_ achieves ZVS conduction. *V*_*AB*_ is *V*_*DC*_/2, and *i*_*r1*_ is positive. *L*_*m1*_ participates is in the resonance, *i*_*r1*_ is equal to *i*_*m1*_, and the transformer does not transmit power to the secondary side. The differential equations of *i*_*r1*_ and *i*_*m1*_ are the same as those of *M1*.

*Interval M8 (t*_*7*_*-t*_*8*_, Figure 4h): In this mode, *Q*_*3*_ is in the conduction state. At time *t*_*7*_, *Q*_*1*_ is turned off. The current on the primary side flows through the parasitic diode of *Q*_*2*_, *V*_*AB*_ decreases from *V*_*DC*_*/2* to *0*, and *i*_*r1*_ is positive. *L*_*m1*_ is clamped at -*nV*_*o*_. *i*_*m1*_ decreases linearly. On the secondary side, *D*_*2*_ is turned on and *I*_*D2*_ rises from zero. *N*_*S12*_, *N*_*S22*_ and *D*_*2*_ constitute the power transmission path of the converter. The differential equations of *i*_*r1*_ and *i*_*m1*_ are:

5 $$\frac{{di_{r1} }}{dt} = \frac{{ - V_{cr1} }}{{L_{r1} }} - \frac{{8n^{2} i_{R} R_{L} }}{{\pi^{2} L_{r1} }}$$

6 $$\frac{{di_{m1} }}{dt} = \frac{{8n^{2} i_{R} R_{L} }}{{\pi^{2} L_{m1} }}$$

*Interval M9 (t*_*8*_*-t*_*9*_, Figure 4i): In this mode, *Q*_*3*_ remains on. At time *t*_*8*_, *Q*_*2*_ achieves ZVS conduction. *V*_*AB*_ is 0, and *i*_*r1*_ is negative. The rest is the same as *M8*.

*Interval M10 (t*_*9*_*-t*_*10*_, Figure 4j): In this mode, *Q*_*2*_ and *Q*_*3*_ are in the conduction state. *V*_*AB*_ is 0, and *i*_*r1*_ is negative. At time *t*_*9*_, *i*_*m1*_ is equal to *i*_*r1*_, *L*_*m1*_ participates is in the resonance. The transformer does not transmit power to the secondary side, *D*_*2*_ realizes ZCS shutdown, and the load relies on the capacitor *C*_*o*_ to provide energy. The differential equations of *i*_*r1*_ and *i*_*m1*_ are:

7 $$\frac{{di_{r1} }}{dt} = \frac{{di_{m1} }}{dt} = \frac{{ - V_{cr1} }}{{L_{m1} + L_{r1} }}$$

### Current sharing principle

As shown in Fig. 2b, the following assumptions are made : (1) The secondary winding groups *N*_*s11*_ and *N*_*s12*_ of the transformer are fully coupled. Similarly, the windings *N*_*s21*_ and *N*_*s22*_ are also fully coupled. They are exactly the same as the coupling coefficient of the primary winding. (2) The secondary side parameters of each phase in the converter are completely symmetrical.

Based on the above assumptions, it can be seen that the voltage and current on *N*_*s11*_ and *N*_*s12*_ are exactly the same. Similarly, the voltage and current on *N*_*s21*_ and* N*_*s22*_ are also exactly the same. At the same time, since *N*_*s11*_ and *N*_*s21*_ are symmetrically connected in series, the current on *N*_*s11*_ and *N*_*s21*_ is symmetrical (the same size, opposite direction). Similarly, the currents on *N*_*s22*_ and *N*_*s12*_ are also symmetrical. Therefore, the primary side current *i*_*p1*_ and *i*_*p2*_ of the two transformers are symmetrical. Since the resonant current *i*_*r1*_ mainly depends on *i*_*p*_. Therefore, the resonant current of the two-phase resonant tank in the proposed converter is almost symmetrical. The above analysis is based on assumptions. However, in practice, the above conclusions cannot be fully realized. Therefore, in the next chapter, the analysis of current sharing performance will consider the influence of transformer leakage inductance mismatch.

### Input capacitor voltage equalization principle

Based on Figs 3 and 4, the input capacitor *C*_*1*_ (*C*_*2*_) is charged (discharged) and discharged (charged) at *M*_*1-2*_ and *M*_*6-7*_, respectively, in a switching cycle. In the modulation strategy of this paper, the duration of *M*_*1-2*_ and *M*_*6-7*_ is the same, so the voltage of *C*_*1*_ and *C*_*2*_ is the same in theory, both of which are *V*_*DC*_ / 2. However, in practice, due to the tolerance between components, it is difficult to ensure the voltage balance between *C*_*1*_ and *C*_*2*_, so it is necessary to adopt an appropriate modulation strategy for the converter to achieve voltage balance. In the modulation strategy adopted in this paper, the duration of *M*_*1-2*_ and *M*_*6-7*_ can be controlled by properly adjusting the phase difference between *Q*_*1*_ and *Q*_*3*_, so as to adjust the charge and discharge time of *C*_*1*_ and *C*_2_, and then realize the voltage balance between the input capacitors.

## Performance analysis

### Wide voltage and current gain range

**Figure 5:**
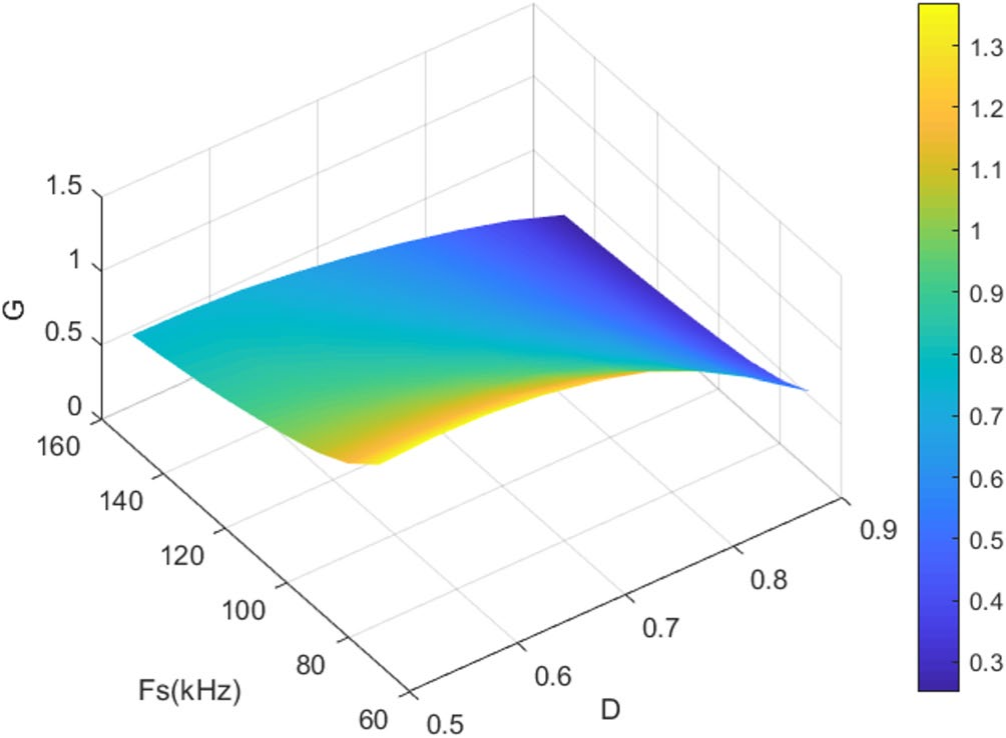
gain margin (The *x*-axis is the duty cycle *D* of the proposed converter, the *y*-axis is the operating frequency *f*_*s*_ of the converter, and the z-axis is the voltage gain *G* of the converter).

The traditional LLC resonant converter usually uses PFM modulation to obtain ZVS in the full load range. However, as the operating frequency increases, its gain gradually slows down, which hinders the LLC resonant converter from achieving a wide gain range. Therefore, the proposed converter adopts a PFM + PWM hybrid modulation strategy to achieve a wide gain range. Among them, PFM is used as the main modulation method to accurately control the output, and PWM is used as an auxiliary modulation method to broaden the gain range of the converter. The specific modulation idea is to control the output to a predetermined range by PWM, and then control the output accurately by PFM. The relationship between duty cycle *d*, operating frequency *f*_*s*_ and voltage gain *G* (*G* = *V*_*o*_ / *V*_*in*_) is shown in the Fig. 5.

### Current sharing performance

In the previous chapter, when the principle of current sharing is given, it is assumed that the secondary winding group of the transformer is completely coupled and the parameters are completely equal. However, in the actual production of the transformer, the assumed content cannot be fully realized. Therefore, in the actual analysis of the current sharing performance of the proposed converter, it is necessary to consider the mismatch of the secondary leakage inductance of the transformer between the phases. Figure 2a shows the equivalent circuit model considering the secondary leakage inductance mismatch of the transformer, where *L*_*s11*_,* L*_*s12*_, *L*_*s21*_ and *L*_*s22*_ represent the secondary leakage inductance of the transformer.

### Define the calculation method of current sharing error

The resonant current sharing error *δ*_*ir*_ and load current sharing error *δ*_*Io*_ are defined as

8 $$\delta_{{i_{r} }} = \left| {\frac{{i_{{r1\left( {rms} \right)}} - i_{{r2\left( {rms} \right)}} }}{{i_{{r1\left( {rms} \right)}} + i_{{r2\left( {rms} \right)}} }}} \right| \times 100\%$$

9 $$\delta_{{I_{o} }} = \left| {\frac{{\left| {i_{o1} } \right| - \left| {i_{o2} } \right|}}{{\left| {i_{o1} } \right| + \left| {i_{o2} } \right|}}} \right| \times 100\%$$

where *i*_*r1(rms)*_, *i*_*r2(rms)*_ and *i*_*o1*_, *i*_*o2*_ are the root mean square and output current of the resonant current of phase 1 and phase 2, respectively. When *δ*_*ir*_ = 0, that mean the resonant current is symmetrically distributed between the two phases, *δ*_*Io*_ = 1 means that only one phase of the converter is in working state.

### Resonance current sharing error analysis

**Figure 6:**
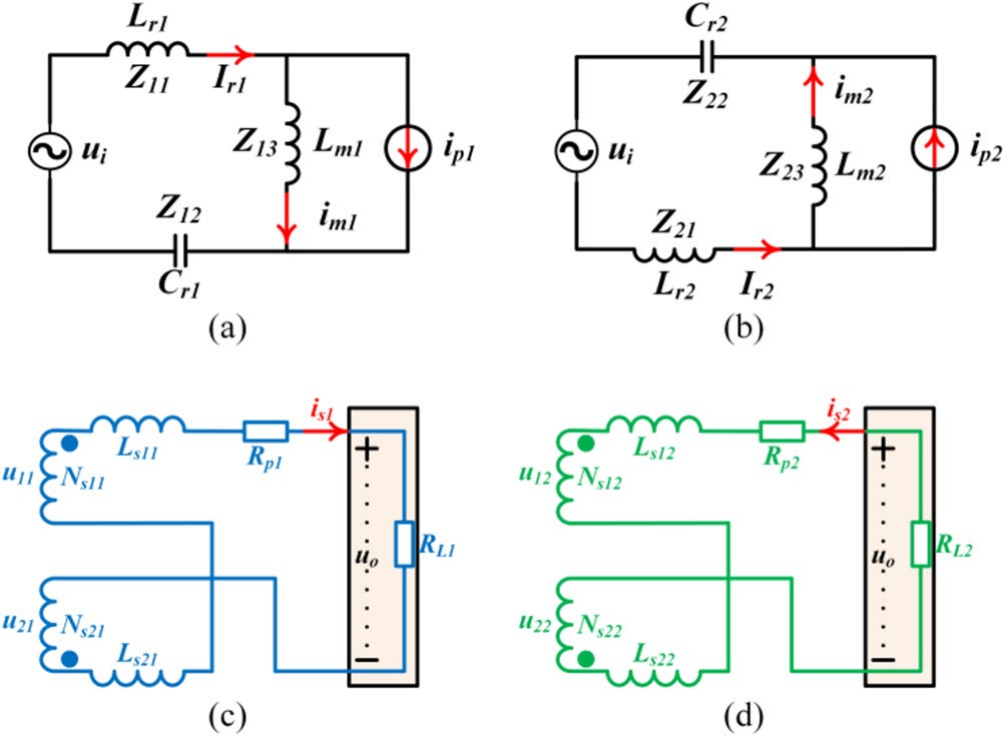
FHA equivalent circuit model. (**a**) The primary side of phase 1. (**b**) The primary side of phase 2. (**c**) The secondary side of phase 1. (**d**) The secondary side of phase 2. (*u*_*i*_ is the equivalent voltage of the primary side, *i*_*p*_ is the current of the primary side of the transformer, *L*_*r*_ is the resonant inductance, *L*_*m*_ is the excitation inductance, *C*_*r*_ is the resonant capacitor, *Z* is the equivalent impedance of the resonant element, *u* is the equivalent input voltage of the secondary side of the transformer, *N* is the winding of the transformer, and *R*_*p*_ is the equivalent resistance of the rectifier part of the converter).

The First Harmonic Approximation (FHA) equivalent circuit of the resonant tank is shown in Fig. 6(a,b). *i*_*p1*_ and *i*_*p2*_ can be expressed as (10). It can be seen from (10) that *i*_*p1*_ and *i*_*p2*_ are always symmetrical. Therefore, we can define *i*_*p1*_ and *i*_*p2*_ as (11).

10 $$\left\{ {\begin{array}{*{20}c} {i_{p1} = \frac{{i_{s1} - i_{s2} }}{n}} \\ {i_{p2} = \frac{{i_{s2} - i_{s1} }}{n}} \\ \end{array} } \right.$$

11 $$i_{p1} = - i_{p2} = i_{p}$$

As shown in Fig. 6, *Z*_*11*_, *Z*_*12*_, *Z*_*13*_, *Z*_*21*_, *Z*_*22*_ and *Z*_*23*_ are the impedances of *L*_*r1*_, *C*_*r1*_, *L*_*m1*_, *L*_*r2*_, *C*_*r2*_ and *L*_*m2*_, respectively. Setting parameters of the first phase as reference variables. The parameters of the second phase have a certain tolerance in the reference. Therefore, the resonant components *L*_*r2*_, *C*_*r2*_, *L*_*m2*_ have deviations based on *L*_*r1*_, *C*_*r1*_, *L*_*m1*_, respectively. The component impedance *Z*_*21*_, *Z*_*22*_, *Z*_*23*_ have corresponding deviations based on *Z*_*11*_, *Z*_*12*_, *Z*_*13*_, respectively. Similarly, the resonant current *i*_*r2*_ has corresponding deviations based on *i*_*r1*_. According to the FHA equivalent circuit model, the reference value of the resonant current is

12 $$i_{r1} = \frac{{u_{i} + Z_{13} i_{p1} }}{{Z_{11} + Z_{12} + Z_{13} }}$$

13 $$\left\{ {\begin{array}{*{20}c} {Z_{11} = j2\pi f_{s} L_{r1} } \\ {Z_{12} = - j\frac{1}{{2\pi f_{s} C_{r1} }}} \\ {Z_{13} = j2\pi f_{s} L_{m1} } \\ \end{array} } \right.$$

The impedance can be calculated by:

According to (13), the impedance deviation of the two-phase resonant element can be calculated as:

14 $$\left\{ {\begin{array}{*{20}c} {dZ_{11} = j2\pi f_{s} \left( {L_{r2} - L_{r1} } \right)} \\ {dZ_{12} = j\left( {\frac{1}{{2\pi f_{s} C_{r1} }} - \frac{1}{{2\pi f_{s} C_{r2} }}} \right)} \\ {dZ_{13} = j2\pi f_{s} \left( {L_{m2} - L_{m1} } \right)} \\ \end{array} } \right.$$

The two phases of the proposed converter are symmetrically interleaved, so *i*_*r1*_ and *i*_*r2*_ are symmetrical. However, there is a tolerance of the resonant element, so there is a deviation between│*i*_*r1*_│and│*i*_*r2*_│, which is used to indicate $\Delta I$. Then the relationship between the resonant currents can be expressed as:

15 $$\Delta I = \left| {i_{r2} } \right| - \left| {i_{r1} } \right|$$

The combination of (12)(13)(14) can transform (15) into:

16 $$\Delta I = \frac{{ - \left( {u_{i} + Z_{13} i_{p} } \right)dZ_{11} }}{{\left( {Z_{11} + Z_{12} + Z_{13} } \right)^{2} }} + \frac{{ - \left( {u_{i} + Z_{13} i_{p} } \right)dZ_{12} }}{{\left( {Z_{11} + Z_{12} + Z_{13} } \right)^{2} }} + \frac{{\left[ {\left( {Z_{11} + Z_{12} } \right)i_{p} - u_{i} } \right]dZ_{13} }}{{\left( {Z_{11} + Z_{12} + Z_{13} } \right)^{2} }}$$

where *u*_*i*_ and *i*_*p*_ are the fundamental amplitude of the resonant tank voltage and the primary side current of the transformer, respectively. *u*_*i*_ and *i*_*p*_ are equal for the two phases of the converter. According to the analysis of the FHA circuit model, it can be calculated as (17), where *V*_*in*_ and *I*_*o*_ are the input voltage and output current of the converter, respectively.

17 $$\left\{ {\begin{array}{*{20}c} {u_{i} = \frac{2}{\pi }V_{in} } \\ {i_{p} = \frac{\pi }{2n}I_{o} } \\ \end{array} } \right.$$

At this time, combined with (8), (15) is transformed into (18), so as to further analyze the primary side resonance current sharing error *δ*_*ir*_.

18 $$\delta_{{i_{r} }} = \left| {\frac{\Delta I}{{2i_{r1} + \Delta I}}} \right| \times 100\%$$

Considering the LLC resonant converter needs to work in the inductive range, under critical conditions, the *δ*_*ir*_ can be calculated by substituting (16) into (18):

19 $$\delta_{{i_{r} }} \le \frac{{\left| {dZ_{11} + dZ_{12} + dZ_{13} } \right|}}{{\left| {\left( {dZ_{11} + dZ_{12} + dZ_{13} } \right) + 2\left( {Z_{11} + Z_{12} + Z_{13} } \right)} \right|}}$$

### Load current sharing error

Figure 6(c,d) give the FHA equivalent circuit model of the secondary side of the transformer, where *R*_*P1*_ and *R*_*P2*_ are the equivalent impedance of the secondary side. According Fig. 6, the secondary current *i*_*s1*_ and *i*_*s2*_ can be calculated as

20 $$\left\{ {\begin{array}{*{20}c} {i_{s1} = \frac{{u_{11} + u_{21} - u_{o} }}{{Z_{s1} }}} \\ {i_{s2} = \frac{{u_{o} - u_{21} - u_{22} }}{{Z_{s2} }}} \\ \end{array} } \right.,u_{11} + u_{21} = u_{21} + u_{22}$$

where *Z*_*s1*_ and *Z*_*s2*_ are:

21 $$\left\{ {\begin{array}{*{20}c} {Z_{s1} = R_{p1} + j2\pi f_{s} \left( {L_{s11} + L_{s21} } \right)} \\ {Z_{s2} = R_{p2} + j2\pi f_{s} \left( {L_{s12} + L_{s22} } \right)} \\ \end{array} } \right.$$

For the convenience of calculation, (9) is transformed into:

22 $$\delta_{{I_{o} }} = \left| {\frac{{i_{s1} + i_{s2} }}{{i_{s1} - i_{s2} }}} \right| \times 100\%$$

Substituting (20) (21) into (22), the *δ*_*Io*_ can be calculated as:

23 $$\delta_{{I_{o} }} = \left| {\frac{{\left( {L_{s12} + L_{s22} } \right) - \left( {L_{s11} + L_{s21} } \right)}}{{\left( {L_{s12} + L_{s22} } \right) + \left( {L_{s11} + L_{s21} } \right)}}} \right| \times 100\%$$

### Low output current ripple

**Figure 7:**
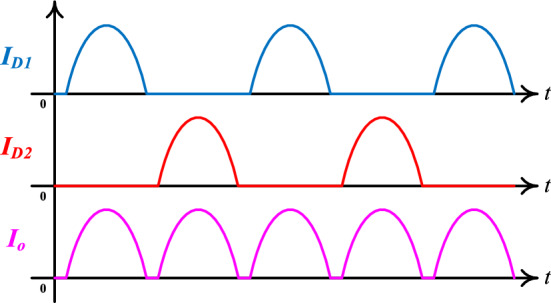
waveform of *I*_*D1*_, *I*_*D2*_ and *I*_*o*_ (The x axis is the time *t*, and the y axis is the output current *I*_*D1*_ of the phase 1, the output current *I*_*D2*_ of the phase 2, and the output current *I*_*o*_ of the proposed converter).

The waveforms of *I*_*D1*_, *I*_*D2*_ and *I*_*o*_ are shown in Fig. 7, where *I*_*o*_ is the output current of the converter. It can be seen from this figure that for single-phase structure, *I*_*o*_ is only equal to one of *I*_*D1*_ or *I*_*D2*_, while the proposed converter uses a symmetrical interleaving technique to make *I*_*o*_ equal to the superposition of *I*_*D1*_ and *I*_*D2*_, so that the amplitude of the output current ripple is reduced by half and the frequency is doubled. Therefore, in order to obtain the same filtering effect, the requirement of the proposed converter for the filter capacitor is reduced by 4 times compared with the single-phase converter. This greatly reduces the design difficulty of the filter capacitor.

### Application extension ability

**Figure 8:**
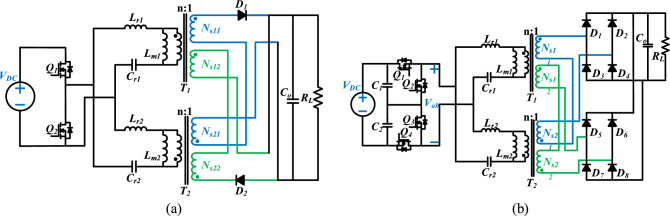
The extended application of the proposed method. (**a**) Half-bridge inverter + full-wave rectifier LLC resonant converter. (**b**) Three-level inverter + full-bridge rectifier LLC resonant converter a shows that the proposed method is applied to the half-bridge inverter + full-wave rectifier LLC resonant converter, which is suitable for low-power applications. (**b**) shows that the proposed method is applied to the three-level inverter + full-bridge rectifier LLC resonant converter, which is suitable for high-power applications).

Based on the three-level inverter + full-wave rectifier LLC resonant converter, the proposed method is analyzed in the paper. However, the method also has good scalability. On the primary side, it can be extended to full-bridge inverter, half-bridge inverter and three-level inverter. In terms of resonant tank, it can be extended to series resonance and parallel resonance. In the secondary side, it can be extended to full-wave rectification, bridge rectification and voltage doubling rectification. In practical applications, the primary side, resonant tank and secondary side of the converter can be arbitrarily combined according to different power requirements. For example, in low power applications, a half-bridge inverter + full-wave rectifier combined LLC converter can be used, as shown in Fig. 8a, while in high power applications, a three-level inverter + full-bridge rectifier combined LLC converter can be used, as shown in Fig. 8b.

### Performance comparison

Table 1 compares the proposed converter with similar converters in terms of component number, modulation mode, output current ripple, current sharing performance and voltage and current gain range to reflect the performance advantages of the proposed converter.

**Table 1:** The performance comparison of the proposed converter with similar converters.

Converter	Proposed converter	Converter in^28^	Converter in^29^	Converter in^30^	Converter in^31^
Number of MOSFETs	4	8	8	4	6
Number of diodes	2	0	4	4	2
Number of tranformers	2	2	2	2	2
Modulation strategy	PFM + PWM	PFM	PFM + PWM	PFM	PFM + PSM
Current sharing method	Passive	None	None	Passive	Active
Output current ripple	Small	Small	Small	High	High
Gain range	Wide	Medium	Wide	Medium	Medium

In order to reflect the fairness of the comparison, the converters summarized in the table are two-phase parallel LLC resonant converters. It can be seen that the proposed converter uses the least number of components in these converters. Concerning output current ripple, the converters in^30,31^ are in ordinary parallel form, which holds no significance for reducing output current ripple, the converters in^28,29^ and the proposed converter adopt interleaved parallel technology to reduce the output current ripple. Concerning current sharing method, in terms of current sharing method, the converter in^28,29^ does not use the current sharing method, which will have a very negative impact on the life and heat dissipation of the converter. The converter in^31^ adopts the active current sharing method. Although this current sharing method is effective, it adds current sensor as auxiliary device in the converter and increases the complexity of the control. The converter in^30^ and the proposed converter adopt the passive current sharing method, which does not add any auxiliary devices and auxiliary control strategies, and has the advantages of low cost and high reliability. Concerning modulation strategy and gain range, the converter in^28,30,31^ adopts PFM modulation strategy, and the gain range in this modulation mode is very limited, while the converter in^29^ and the proposed converter adopt PFM + PWM hybrid modulation strategy, which greatly broadens the gain range of the converter.

## Experimental verification

### Experimental setup

**Figure 9:**
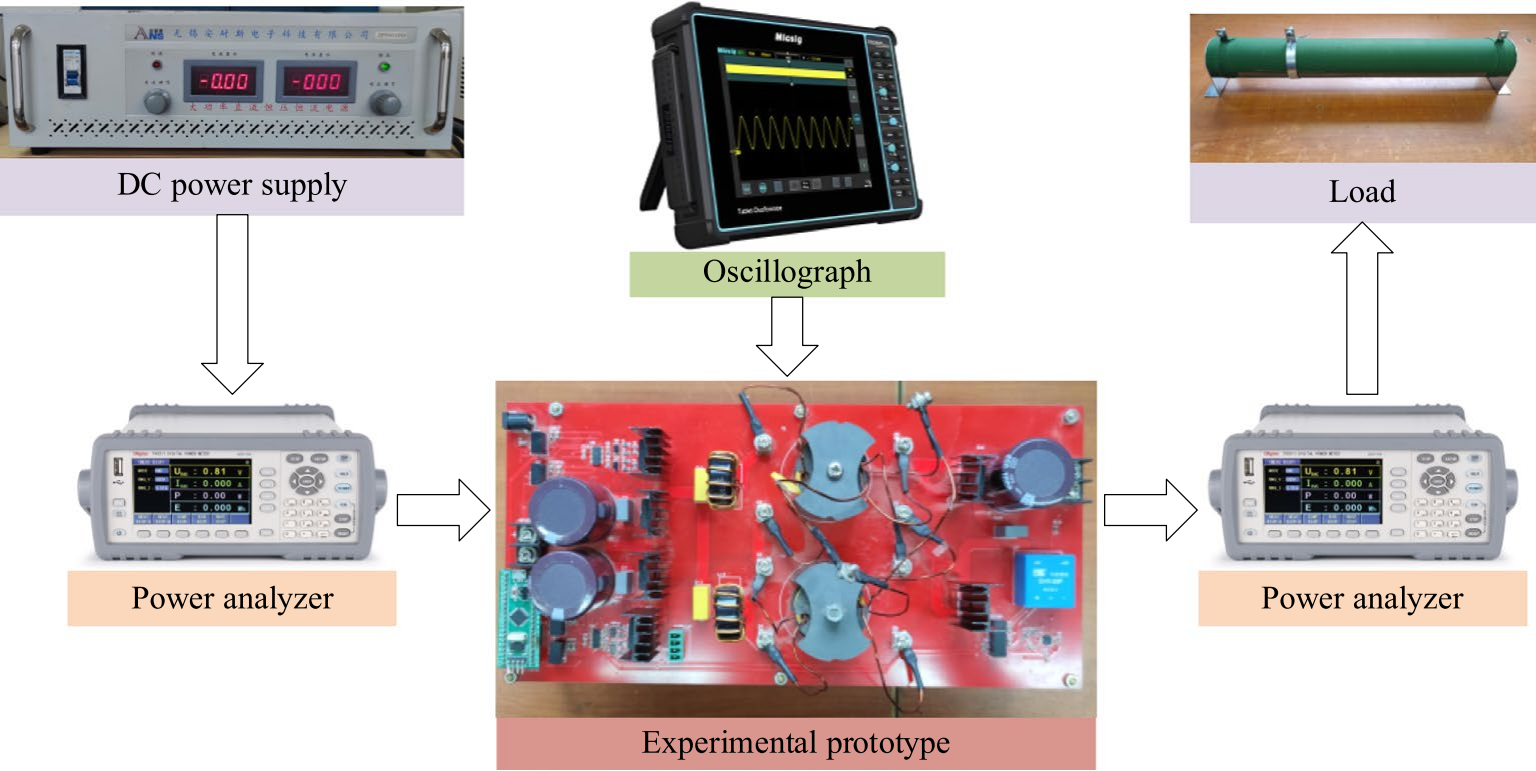
Experimental platform. (The instruments used in the experimental platform have been marked out in the figure).

In order to verify the above theoretical analysis, this article constructed an experimental prototype, and Fig. 9 shows the experimental platform diagram. Its detailed parameters are shown in Table 2.

**Table 2:** Detailed parameters of the proposed converter.

Component	value
Input voltage (*V*_*DC*_)	100 V
Turns ratio (*n:1*)	1:1
Output power (*P*_*out*_)	600W
Resonant frequency (*fr*)	100 kHz
MOSFETs (*Q*_*1-4*_)	IPW60R019C7
Diode (*D*_*1*_, *D*_*2*_)	C5D50065D
Resonant capacitance (*C*_*r1*_, *C*_*r2*_)	390 nF
Resonant inductance (*L*_*r1*_, *L*_*r2*_)	6.46 μH
Transformer leakage inductance	Phase1: 2.1μH
	Phase2: 2.1μH
Magnetizing inductance (*L*_*m1*_, *L*_*m2*_)	25.84 μH
Driver IC	ADuM3223ARZ
MCU	STM32F334C8T6

### Experimental results

In this section, the driving signal of *Q*_*1*_-*Q*_*4*_, resonant tank voltage (*V*_*ab*_) and resonant current (*i*_*r2*_) are measured to verify the working principle of the proposed converter. At the same time, the gate-source voltage (*V*_*G1*_, *V*_*G2*_, *V*_*G3*_, *V*_*G4*_), drain-source voltage (*V*_*S1*_, *V*_*S2*_, *V*_*S3*_, *V*_*S4*_), and resonant current (*i*_*r1*_, *i*_*r2*_) were measured to verify the soft-switching conditions. In addition, the load current (*i*_*D1*_, *i*_*D2*_) and resonant current (resonant element tolerance is 10%) of the two phases of the converter under different loads, and the dynamic waveform of the resonant current under variable load conditions are also measured to verify the current sharing performance. At the same time, the output voltage at different operating frequencies and duty cycles is also measured to verify the wide voltage gain range. Finally, the efficiency curve of the converter is given. The experimental system includes a DC power supply (JP50010D), two voltage probes with an oscilloscope (STO1004), an AC current probe (CP9012S) with an oscilloscope, a resistance load and two power meters (TH3331).

**Figure 10:**
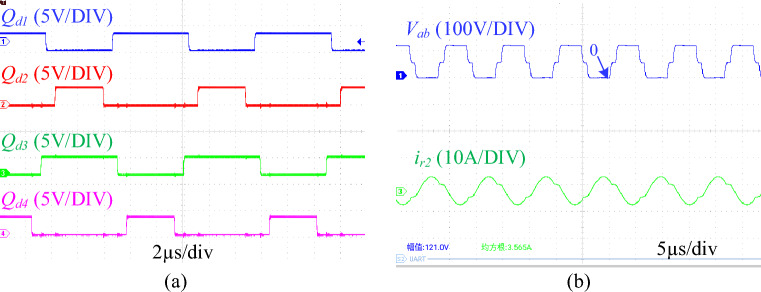
The key waveforms of the proposed converter. (**a**) Drive signals for *Q*_*1*_-*Q*_*4*_. (**b**) The waveforms of *V*_*ab*_ and *i*_*r2*_. (*Q*_*d1*-*d4*_ is the driving signal of *Q*_*1*-*4*_ measured on the single chip microcomputer, *V*_*ab*_ is the resonant tank voltage, and *i*_*r2*_ is the resonant current of 2 phases).

Figure 10a shows the driving signal waveforms of* Q*_*1*_-*Q*_*4*_ at *d* = 0.6, where *Q*_*d1*_-*Q*_*d4*_ are the driving signals of *Q*_*1*_-*Q*_*4*_ measured from the single-chip microcomputer, and Fig. 10b shows the resonant tank voltage *V*_*ab*_ and the resonant current *i*_*r2*_ of the proposed converter under the driving signal. It can be seen from Fig. 10 that the timing of the relevant steady-state waveform is consistent with the theoretical analysis in Fig. 3.

**Figure 11:**
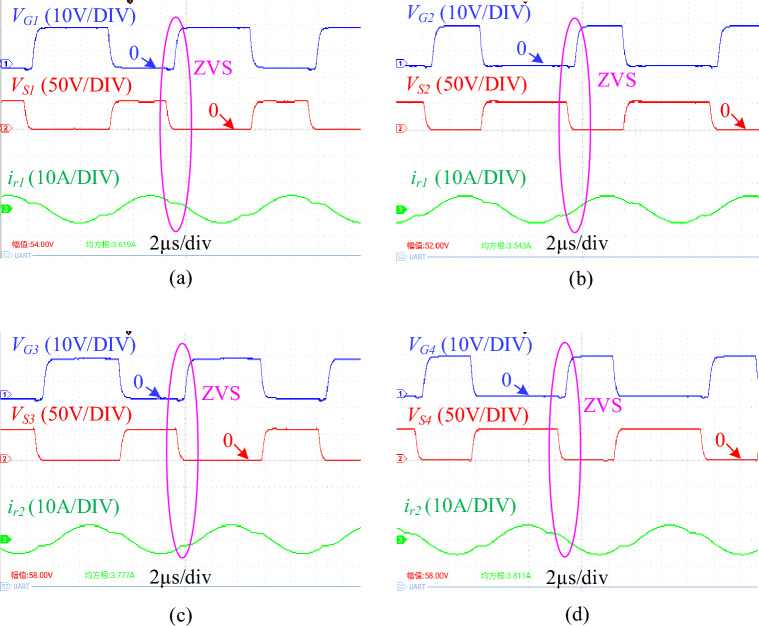
Experimental switching waveforms. (**a**) Switching waveforms of Q1. (**b**) Switching waveforms of Q2. (**c**) Switching waveforms of Q3. (**d**) Switching waveforms of Q4. (*V*_*G*_ is the gate-source voltage of MOSFET, *V*_*S*_ is the drain-source voltage of MOSFET, and *i*_*r*_ is the resonant current).

The switching waveforms of *Q*_*1*_, *Q*_*2*_, *Q*_*3*_ and *Q*_*4*_ at a duty cycle of 0.6 are shown in Fig. 11. As shown in the figure, before the gate-source voltage (*V*_*G1*_, *V*_*G2*_, *V*_*G3*_, *V*_*G4*_) of the four MOSFETs rises, the drain-source voltage (*V*_*S1*_, *V*_*S2*_, *V*_*S3*_, *V*_*S4*_) has dropped to zero, that is, there is no overlap between the driving signal and the drain-source voltage. The ZVS conditons of transistors are obtained. At the same time, this switching transient occurs when the resonant tank enters the ternary resonant state (the resonant current enters a gentle change state).

To evaluate the current sharing performance, a 10% difference is introduced in the parameters of the resonant elements in the two phases during testing. Specific parameters are outlined in Table 3.

**Table 3:** The detailed parameters of the resonant elements in the two phases.

Input voltage	100 V
Resonant capacitance	Phase 1: 387nF
	Phase 2: 418nF (1.1C_r1_)
Resonant inductance	Phase 1: 6.7μH
	Phase 2: 7.3μH (1.1L_r1_)
Magnetizing inductance	Phase 1: 24.8μH
	Phase 2: 26.9μH (1.1L_m1_)

**Figure 12:**
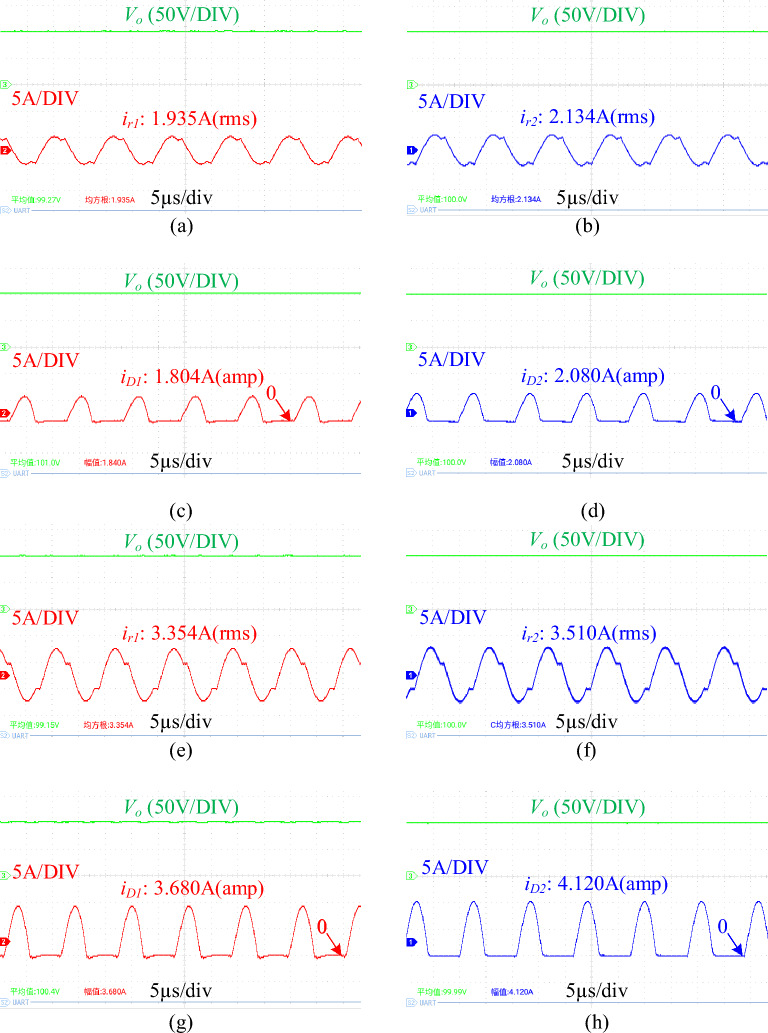
key current waveform at different load. (**a**) phase 1 resonant current at 50% load. (**b**) phase 2 resonant current at 50% load. (**c**) phase 1 load current at 50% load. (**d**) phase 2 load current at 50% load. (**e**) phase 1 resonant current at full load. (**f**) phase 2 renonant current at full load. (**g**) phase 1 load current at full load. (**h**) phase 2 load current at full load. (*V*_*o*_ is the output voltage, *i*_*r*_ is the resonant current, *i*_*D*_ is the load current).

**Figure 13:**
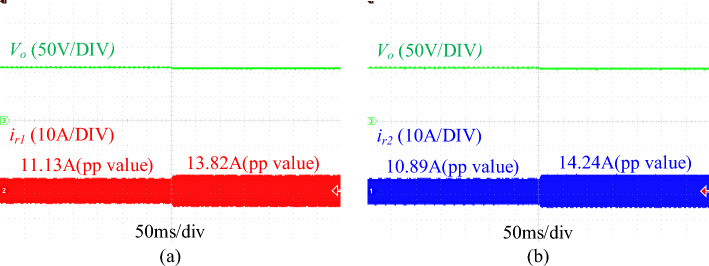
Dynamic waveforms of *i*_*r1*_ and* i*_*r2*_ under variable load. (**a**) Dynamic waveform of *i*_*r1*_. (**b**) Dynamic waveform of *i*_*r1*_. (*V*_*O*_ is the output voltage, *i*_*r1*_ and* i*_*r2*_ are the resonant current).

Figure 12 shows the waveforms of resonant current and load current under different loads. As shown in the figure, the error of resonant current and load current can be calculated to be 9.3% and 11.5% respectively at half load, and 4.44% and 10.6% respectively at full load. In addition, Fig. 13 shows the dynamic waveform of the resonant current under variable load. It can be seen that the proposed converter still has good current sharing performance even if the load changes suddenly.

**Figure 14:**
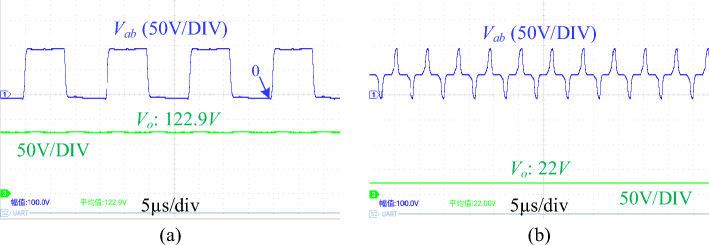
waveforms of *V*_*ab*_ and *V*_*o*_. (**a**) The operating frequency is 60 kHz, and the duty cycle is 0.5. (**b**) The operating frequency is 160 kHz, and the duty cycle is 0.8. (*V*_*ab*_ is the resonant cavity voltage, *V*_*o*_ is the output voltage).

Figure 14 shows output voltage waveforms at different duty cycles and operating frequencies. The input voltage is fixed to 100 V. When the operating frequency is 60 kHz and the duty cycle is 0.5, the output voltage is 122.9 V. When the operating frequency is 160 kHz and the duty cycle is 0.8, the output voltage is 22 V. This is consistent with the analysis results in Fig. 5, which verifies the ability of the proposed converter to have a wide range of voltage and current gain.

**Figure 15:**
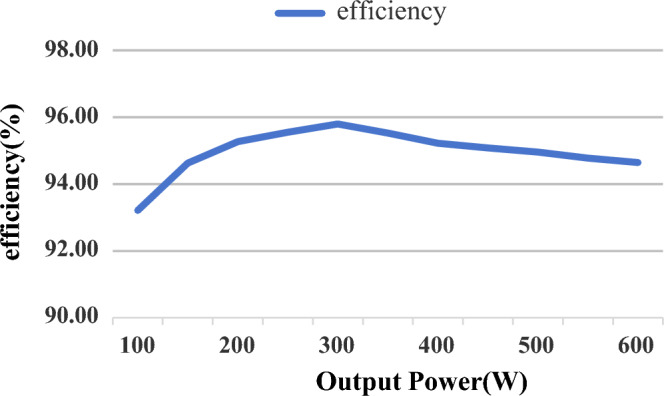
The efficiency of the proposed converter (The *x*-axis is the output power of the converter and the efficiency of the *y*-axis converter).

The efficiency curve of the proposed converter at the operating frequency of 100 kHz is shown in Fig. 15. It can be seen that the peak efficiency is 95.8%.

## Conclusion

This paper proposed a single-bridge interleaved three-level LLC resonant converter with current sharing capability. Providing an in-depth analysis of its working principle. Experimental verification is conducted with a 600 W prototype. Through measurements such as soft-switching waveforms, resonant current, and load current waveforms (with a 10% tolerance in resonant elements), output voltage waveforms, and efficiency curves, it is confirmed that the proposed converter exhibits excellent current-sharing ability, a widely voltage regulation range, low output current ripple, and high efficiency. These advantages position the proposed converter as a good choice in fuel cell system applications.

## Data Availability

This article contains all the data generated by this study.
